# A Comparative Study of Sublingual Misoprostol Versus Intramuscular Oxytocin in the Active Management of Third Stage of Labor

**DOI:** 10.7759/cureus.33339

**Published:** 2023-01-04

**Authors:** Shweta Mishra, Sarita Tirkey, Anupa Prasad, Kiran Trivedi

**Affiliations:** 1 Obstetrics and Gynaecology, Rajendra Institute of Medical Sciences, Ranchi, IND; 2 Biochemistry, Rajendra Institute of Medical Sciences, Ranchi, IND

**Keywords:** postpartum hemorrhage, third stage of labor, uterotonics, sublingual misoprostol, oxytocin

## Abstract

Objective

Misoprostol has attracted low-income low-resource countries for the active management of the third stage of labor. The objective of this study was to compare the efficacy of sublingual misoprostol and intramuscular oxytocin in the active management of the third stage of labor.

Study design

This was a prospective randomized controlled trial in which a total of 407 healthy pregnant women having singleton pregnancy, cephalic presentation, and normal vaginal delivery were divided into two groups. In the first group (n=203), women received 600 µg misoprostol tablet sublingually, and in the second group (n=204), women received 10 IU of intramuscular oxytocin, within 1 minute of the delivery of the baby during the third stage of labor. Three patients from the first group and four patients from the second group were excluded from the analysis due to traumatic postpartum hemorrhage (PPH). The primary outcome was an incidence of PPH. Secondary outcomes were the duration of the third stage of labor, amount of blood loss, fall in hemoglobin concentration after 48 hours of delivery, need for additional uterotonics, and side effects of the drugs. Data were compared using the chi-square and independent samples t-test.

Results

The incidence of PPH was 6.5% in the misoprostol group as compared to 2% in the oxytocin group (p=0.026). The misoprostol group also had significantly higher blood loss (293.75±125.8 mL) and a greater fall in hemoglobin level (0.58±0.25 g/dL) as compared to that in the oxytocin group (226.13±98.44 mL and 0.45±0.20 g/dL) (p<0.001). The mean duration of the third stage of labor was significantly higher in the misoprostol group (5.31±2.1 min) as compared to that in the oxytocin group (3.65±1.75 min) (p<0.001). The additional need for uterotonics was recorded in 15% of the study participants in the misoprostol group as compared to 8% in the oxytocin group (p=0.028). The incidence of side effects such as shivering and fever was significantly higher in the misoprostol group as compared to the oxytocin group. No significant difference between the two groups was observed concerning the incidence of nausea, vomiting, diarrhea, and headache.

Conclusion

Intramuscular oxytocin is a safe and useful alternative to sublingual misoprostol in facilitating the third stage of labor with minimal blood loss, fewer incidences of hemorrhage, and fewer adverse effects.

## Introduction

The World Health Organization recommends the use of uterotonics for the prevention of postpartum hemorrhage (PPH) during the third stage of labor in all types of birth [1]. According to these guidelines, administration of a uterotonic immediately after the birth of the baby is useful in reducing the risk of PPH. Misoprostol, carboprost, and oxytocin are some of the commonly used uterotonics for the management of the third stage of labor. The use of prostaglandins such as misoprostol is popular as it is economical and can be administered orally, sublingually, and through vaginal or rectal routes, whereas oxytocin can be delivered intravenously or intramuscularly. Oxytocin is a hormone that stimulates uterine contractions and prevents PPH and is a recommended standard of care during the third stage of labor for the prevention of PPH [1]. However, the use of oxytocin is not popular in less-developed and low-resource settings with frequent electricity power failures as oxytocin needs to be stored under a certain temperature requiring cold-chain maintenance. Moreover, intravenous/intramuscular administration of oxytocin requires the use of sterile syringes and needles [2,3]. In low-resource settings, synthetic prostaglandins such as misoprostol, which also have uterotonic properties, have become popular as they are not only less expensive as compared to oxytocin and carboprost but also their storage and administration do not require maintenance at any specific temperature or need for syringes, needles, or skilled paramedics [4]. It has also gained acceptance as a new and underutilized technology to reduce maternal mortality [5]. Despite its popularity, it is not a well-accepted alternative as it is often considered to provide inadequate effect, forcing the requirement of additional uterotonics and increased risk of PPH [6]. Moreover, misoprostol also carries an increased risk of adverse effects such as maternal fever, chills, nausea, vomiting, diarrhea, and abdominal pain [7]. Despite these limitations, misoprostol has always attracted low-income low-resource countries for the management of the third stage of labor. It is routinely being used in the rural population of India. Some previous studies in India have found it to be as effective as oxytocin for the active management of the third stage of labor [8-9]. However, newer emerging evidence raises questions regarding the safety profile of misoprostol use in the management of the third stage of labor [10]. Considering these conflicting shreds of evidence, the present study was planned to carry out a comparative assessment of sublingual misoprostol versus intramuscular oxytocin in the active management of the third stage of labor.

## Materials and methods

This prospective open-label randomized clinical trial was carried out in the labor room of the Department of Obstetrics and Gynecology at the Rajendra Institute of Medical Sciences (RIMS), Ranchi, from April 2017 to October 2018.

Study population

The inclusion criteria were low-risk pregnant women in labor having singleton pregnancy, cephalic presentation, scheduled to undergo vaginal delivery, and willing to participate in the study. The exclusion criteria were women undergoing instrumental delivery, women with a history of uterine surgery, polyhydramnios, cardiac diseases, respiratory diseases, coagulation disorders, and hypertensive disorder with pregnancy, and women with hypersensitivity to the drugs being administered. Women who had traumatic PPH were also excluded.

Sample size

Sample size calculation was done based on a study by Atukunda et al. [11], who reported that after 24 hours of delivery, primary PPH occurred in 29% of participants in the misoprostol group as compared to 17% of participants in the oxytocin group. Taking into consideration a 95% confidence interval and 80% power of the study, the sample size came to be 197 in each group. However, 200 participants were taken in each group.

Sampling technique

**Figure 1:**
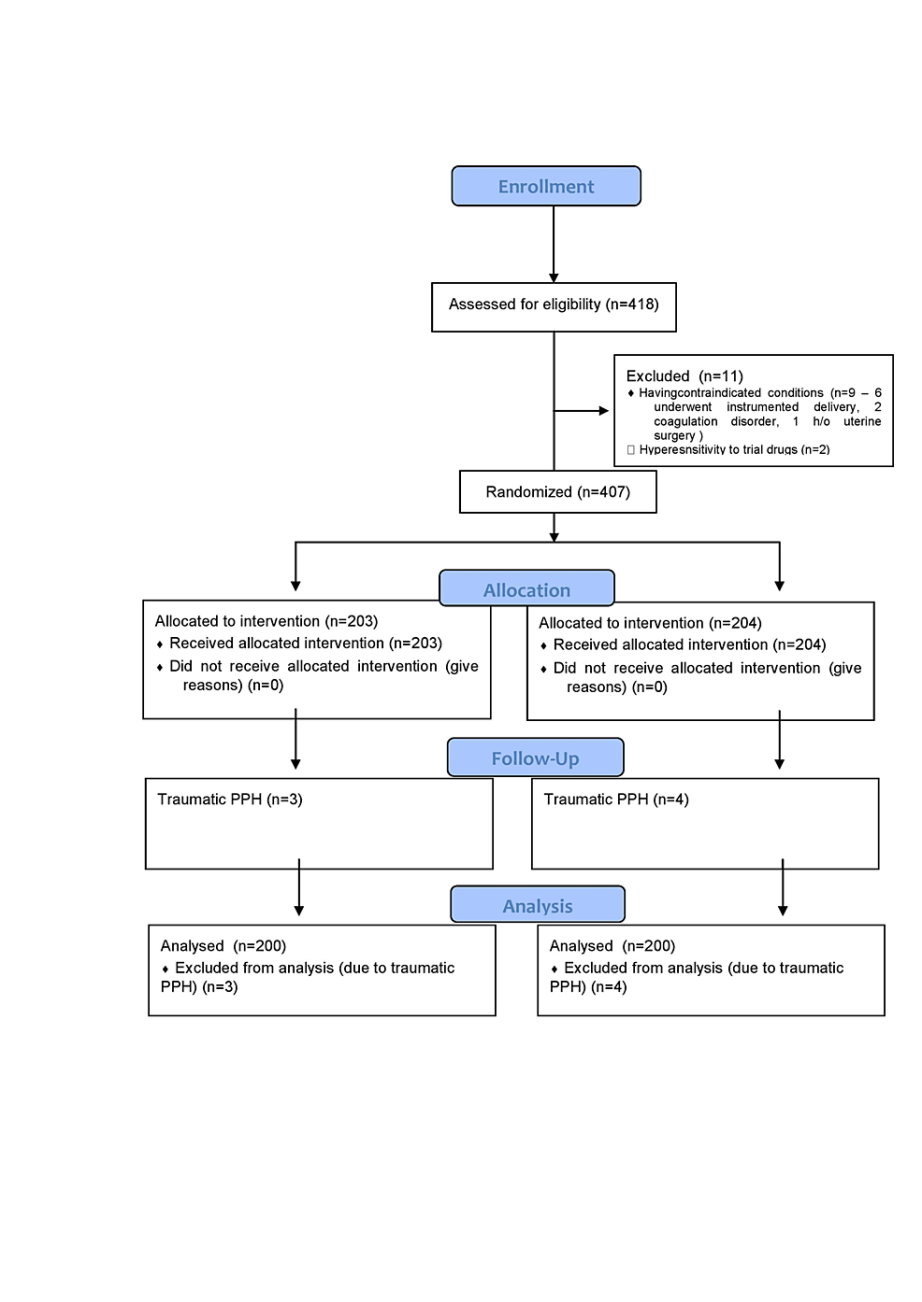
CONSORT flow diagram depicting patient enrollment PPH, postpartum hemorrhage; CONSORT, Consolidated Standards of Reporting Trials

A total of 407 participants were randomized into two groups using computer-generated random number tables. The first group received 600 µg of misoprostol tablet sublingually within 1 minute of delivery of the baby, while the other group received 10 IU of intramuscular oxytocin within 1 minute of delivery of the baby. Active management of the third stage of labor is done for the prevention of PPH. Three cases from the first group and four patients from the second group were excluded due to bleeding from tears and lacerations (traumatic PPH) (Figure 1).

Ethical consideration

Ethical approval was taken from Institutional Ethics Committee, RIMS, Ranchi, vide approval letter number IAEC/IEC28/2017, and written informed consent was obtained from the study participants. Finally, the data were analyzed for 400 participants (200 each in the sublingual misoprostol and intramuscular oxytocin groups, respectively).

Data collection

Routine hematological investigations were conducted including hemoglobin at the time of admission and 48 hours after delivery. When vaginal delivery was imminent, episiotomy was performed if required. The placenta was delivered by controlled cord traction and carefully examined. Additional need for uterotonic drugs (if any) was noted. Within a minute of delivery, linen soiled with amniotic fluid was removed, a new disposable absorbent linen sheet was placed under the woman, and a sterile kidney tray was placed against the women’s buttocks to collect all blood. After stitching episiotomy, the pads and linen were removed when needed. blood loss was measured by weighing all the soaked drapes and pads and subtracting it from the initial dry weight to give an account of the approximate volume of blood in milliliters (1 gram difference in weight was considered equivalent to 1 mL of blood loss). Blood loss was measured for 1 hour after delivery.

Patients’ vitals were monitored for 4 hours after delivery. In atonic PPH, bimanual massage of the uterus was done, uterotonics were given, and patients were managed according to their needs.

Data analysis

Data analysis was conducted using Statistical Package for Social Sciences (SPSS) Version 21 (IBM Corp., Armonk, NY). Categorical/qualitative data are represented as numbers (frequencies) and percentages (proportions), and continuous data are represented as mean (±standard deviation). The chi-square test was used for the comparison of proportional data, while continuous data were analyzed using independent samples t-test. Probability of chance error allowance was kept as 5%; hence, a p-value of less than 0.05 was considered to depict a statistically significant difference.

## Results

The mean age of women was 23.32 ± 3.89 in the misoprostol group and 23.34 ± 3.38 years in the oxytocin group. The majority of them were unbooked (no antenatal checkup) in both the misoprostol (64%) and oxytocin (62.5%) groups. There was a predominance of women from a rural background in both the misoprostol (74.5%) and oxytocin (69.5%) groups. Statistically, no significant difference was observed between the two groups with respect to age, booking status, or residence (p > 0.05) (Table 1).

**Table 1:** Comparison of the demographic profile of the study participants

Characteristic	Misoprostol group (n=200)	Oxytocin group (n=200)	P-value
Mean age ±SD (years)	23.32±3.89	23.34±3.38	0.792
Unbooked participants	128 (64%)	125 (62.5%)	0.756
Rural background	149 (74.5%)	139 (69.5%)	0.265

The mean duration of the third stage of labor was significantly longer in the misoprostol group (5.31 ± 2.1 min) than in the oxytocin group (3.65 ± 1.75 min) (p < 0.001). The mean amount of blood loss was 293.75 ± 125.8 mL in the misoprostol group and 226.13 ± 98.44 mL in the oxytocin group (p < 0.001). The mean fall in hemoglobin level was 0.58 ± 0.25 g/dL in the misoprostol group and 0.45 ± 0.20 g/dL in the oxytocin group (p < 0.001). The incidence of PPH was 6.5% in the misoprostol group and 2% in the oxytocin group (p = 0.026). Additional uterotonics were required in 15% women in the misoprostol group as compared to 8% in the oxytocin group (p = 0.028) (Table 2).

**Table 2:** Comparison of different outcome variables between the two study groups Hb, hemoglobin; PPH, postpartum hemorrhage

Serial number	Outcome	Misoprostol group (n=200)	Oxytocin group (n=200)	P-value
1.	Incidence of PPH	13 (6.5%)	4 (2%)	0.026
2.	Mean amount of blood loss±SD (mL)	293.75±125.8	226.13±98.44	<0.001
3.	Mean fall in Hb±SD (g/dL)	0.58±0.25	0.45±0.20	<0.001
4.	Mean duration of third stage of labor±SD (min)	5.31±2.1	3.65±1.75	<0.001
5.	Need for additional oxytocics	30 (15%)	16 (8%)	0.028

**Figure 2:**
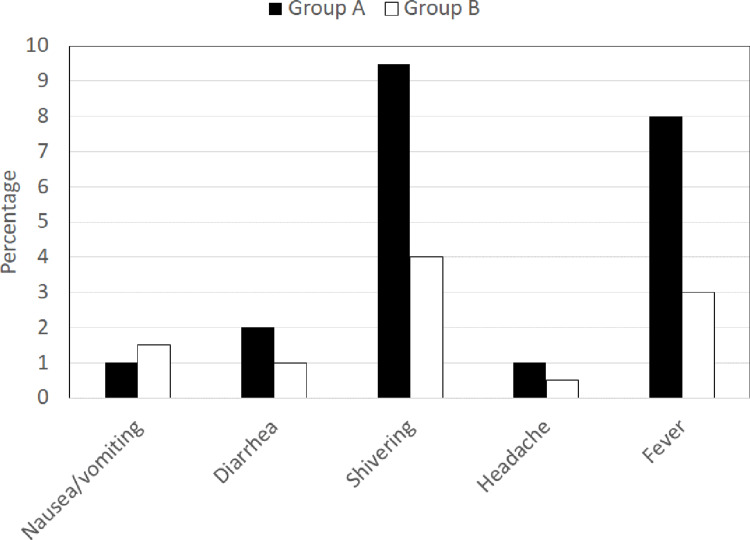
Comparison of the side effects of two drugs used in the study

Side effects such as nausea, vomiting, diarrhea, shivering, headache, and fever were noted in two (1%), four (2%), 19 (9.5%), two (1%), and 16 (8%) women in the misoprostol group, respectively, compared to three (1.5%), two (1%), eight (4%), one (0.5%), and six (3%) women in the oxytocin group, respectively. A significant difference between the two groups was observed for shivering and fever (p < 0.05). Both of these events were significantly higher in the misoprostol group than in the oxytocin group (Figure 2).

## Discussion

This study shows that the mean age was 23.32 years in the misoprostol group and 23.34 years in the oxytocin group. The majority of women were of unbooked status and rural background. This study demonstrates that sublingual misoprostol is not as effective as intramuscular oxytocin for the active management of the third stage of labor. The incidence of PPH was significantly higher in the misoprostol group as compared to the oxytocin group. We found the duration of the third stage of labor to be significantly longer and the amount of blood loss and fall in hemoglobin level to be greater in the misoprostol group than in the oxytocin group. We also witnessed that the need for additional uterotonic drugs, and side effects were significantly higher in the sublingual misoprostol group than in the intramuscular oxytocin group. Thus, the findings in the present study do not support the proposition that misoprostol is as effective as oxytocin for the management of the third stage of labor (proposed by several previous workers) [8,9]. Researchers have highlighted adverse effects as a limitation of the misoprostol group as compared to the oxytocin group. The misoprostol group experienced side effects (i.e. fever, shivering) in significantly more cases [10-12]. Even the studies that found sublingual misoprostol to be as effective as intramuscular oxytocin documented a significantly higher rate of adverse events in the misoprostol group compared to the oxytocin group [8,9]. Mukta and Sahay reported the mean amount of blood loss to be higher (15.9% higher) in the misoprostol group compared to the oxytocin group, but they did not find this difference to be statistically significant [8]. They also observed that the mean fall in hemoglobin levels was higher in the misoprostol group (0.55 g/dl) than in the oxytocin group (0.48 g/dL) but again did not find it to be statistically significant. In the present study, we observed similar results and found this difference to be statistically significant. The reason for this could probably be attributed to the difference in effect size; the present study’s sample size was larger (n = 400) than that of Mukta and Sahay and Singhal et al. (n = 200) [8,9]. Moreover, they found the incidence of PPH to be 8% in the misoprostol group as compared to 6% in the oxytocin group, as well as the additional need for uterotonic drugs to be higher in the misoprostol group (22%) compared to the oxytocin group (16%) [8]. Studies with larger sample sizes, such as the one conducted by Atukunda et al., have highlighted the modest benefit of oxytocin compared to misoprostol, which is in line with the observations in the present study [3]. In the aforementioned study, the risk of PPH was significantly higher in the misoprostol group compared to the oxytocin group; however, the study did not find a statistically significant difference between the two groups. The requirement of additional uterotonics, duration of the third stage of labor, and hemoglobin changes were similar in both groups. The study also reported the rate of adverse events (nausea, vomiting, fever, and shivering) to be significantly higher in the misoprostol group than in the oxytocin group.

A Cochrane review evaluating the use of prostaglandins for the prevention of PPH, which included data from 72 trials involving 52,678 women, concluded that the use of misoprostol over conventional injectable uterotonics cannot be preferred as part of the management of the third stage of labor, particularly in low-risk women [6]. In the present study, we also found that sublingual misoprostol was significantly less effective in preventing PPH than the conventional uterotonic (intramuscular oxytocin) recommended for the active management of the third stage of labor.

Most of the previous evidence emerging from developing low-resource economies such as India, Pakistan, and Nepal seem to be primarily enthusiastic with the low cost, ease of storage, and ease of administration associated with misoprostol use, and they have encouraged its use in pilot studies conducted with inadequate sample sizes. Despite encountering higher rates of blood loss, falls in hemoglobin levels, PPH, and adverse events, they termed misoprostol “as effective as oxytocin.” In their study from Nepal, which had a sample size of 120 (60 in each group), Kaudel et al. observed that in few women blood loss was >200 mL in third stage of labor. Four women in the misoprostol group and one woman in the oxytocin group had > 200 mL of blood loss. Average blood loss was higher (118 mL) in the third stage of labor in the oxytocin group compared to the misoprostol group (115.5 mL), and fall in hemoglobin level was higher in the misoprostol group (0.56±0.35 g/dL) compared to the oxytocin group (0.46±0.29 g/dL) [13]. There was a significant difference in the side effect profile between the two groups. However, they found misoprostol to be as effective as oxytocin.

In a study from Pakistan with a sample size of 70 (35 in each group) conducted by Aziz et al., the median blood loss in the misoprostol group was nearly 100 mL higher than in the oxytocin group. Yet, they did not find this to be statistically significant [12]. This, again, might be due to the smaller sample size. The incidence of side effects was also significantly higher in the misoprostol group than in the oxytocin group.

Studies from other underdeveloped and developing countries report similar findings [14,15], despite showing the higher amount of blood loss, the need for additional uterotonics, and the adverse effects in the misoprostol group. They have mainly focused on statistical differences in a smaller sample size scenario. Pyrexia was found in almost all studies, but Sringamwong et al. found it to be dose dependent and used different doses of misoprostol in their study and reported less percentage of pyrexia in groups having lower doses of misoprostol [16].

Thus, the findings of the present study highlight that sublingual misoprostol cannot be considered as effective as intramuscular oxytocin unless infrastructural lapses are driving decision-making. Most of the previous studies suffer from inadequate sample sizes, and their conclusions should be viewed with caution.

There are several limitations to this study. Firstly, we did not assess neonatal safety and outcomes in our study, which could contribute to the safety profiles of misoprostol and oxytocin. Second, we did not include complicated deliveries, cesarean deliveries, or those who needed induction of labor. Third, this study was a single-centered study. Having multi-centric data could have made the analysis much more representative. Furthermore, a double-blind, randomized controlled trial with a larger sample size and that includes complications during pregnancies, labor, and neonatal outcomes should be conducted. Systematic review and meta-analysis should be carried out to assess maternal and fetal outcomes of misoprostol use during the third stage of labor.

## Conclusions

In the present study, sublingual misoprostol 600 mcg was found to be less effective than intramuscular oxytocin in the active management of the third stage of labor. The results show the risk of increased blood loss, a prolongation of the third stage of labor, a need for additional uterotonics, a higher risk of PPH, and an increased frequency of adverse effects. The use of misoprostol is particularly important for resource-limited settings, where the availability and storage of heat-susceptible oxytocin are challenging.
